# Prevalence and associated risk factors for anaemia amongst pregnant women attending three antenatal clinics in Eswatini

**DOI:** 10.4102/phcfm.v14i1.3339

**Published:** 2022-04-25

**Authors:** Rumbidzai C. Dodzo, Ropo E. Ogunsakin, Themba G. Ginindza

**Affiliations:** 1 Discipline of Public Health Medicine, School of Nursing and Public Health, College of Health Sciences, University of KwaZulu-Natal, Durban, South Africa; 2 Ministry of Health, Eswatini

**Keywords:** anaemia, pregnant women, prevalence, risk factors, Eswatini

## Abstract

**Background:**

Anaemia is a global health problem affecting about a third of the world’s population. In pregnancy, it is a public health concern with consequences for mothers and infants, including maternal death and infant mortality. In low-income countries (LICs), 25% indirect maternal mortality and 30% neonatal deaths are due to anaemia in pregnancy.

**Aim:**

This study aimed to determine the prevalence and risks associated with anaemia amongst pregnant women attending antenatal clinic (ANC) in three health facilities in Eswatini.

**Setting:**

This study was conducted in three health facilities in Eswatini, namely Mankayane, Raleigh Fitkin Memorial (RFM) and Mbabane Hospital.

**Methods:**

This cross-sectional study used non-probability sampling in three hospitals of Eswatini, to select 550 pregnant women, aged 15–49 years. Data were collected from January to March 2021, using face-to-face interviews with a structured questionnaire. Logistic regression was used for statistical analysis.

**Results:**

A total of 550 pregnant women were included in the study. Anaemia prevalence amongst pregnant women was 43.1% with mild, moderate and severe cases of 21.3%; 21.1% and 0.7%, respectively. Prevalence was high amongst women aged 15–19 years (53.3%). Factors associated with anaemia included living in urban areas (odds ratio [OR]: 1.8; confidence interval [CI]: 1.19–2.72), having anaemia 6 months before pregnancy (OR: 4.64; CI: 1.15–18.71), and gestational age at first ANC: third trimester (OR = 10.42; CI: 4.27–25.4) and second trimester (OR: 1.62; CI: 1.02–2.60).

**Conclusion:**

Anaemia remains prevalent amongst pregnant women in Eswatini. A comprehensive anaemia prevention programme would be justified and could lower the country’s burden of anaemia.

## Introduction

Anaemia is a significant public health concern, affecting about two billion people worldwide, amongst which 56 million are pregnant women.^1,2^ Globally, anaemia has been estimated to affect about 800 million children and women. Adolescent girls and women are at a higher risk because of menstruation and the high demand for metabolism during pregnancy.^3^ About 42.0% of pregnant women are affected by anaemia worldwide.^4^ In Southeast Asia, the prevalence of anaemia amongst pregnant women is 48.0% and anaemia in pregnancy causes half of the global maternal deaths. India contributes to 80% of these deaths.^5,6^ South America has the lowest prevalence of 24.1%.^6^ In Africa, studies have found an anaemia prevalence of 57.1% amongst pregnant women.^2^ Previous studies conducted in Tanzania have shown a varying prevalence of anaemia amongst pregnant women, ranging from 18.0% to 68.0%.^7^ The overall prevalence of anaemia amongst pregnant women was 41.0% in Ethiopia and 45.0% in Ghana.^8,9^ Although there is limited data for Southern Africa, a study in South Africa found a prevalence of 42.7%.^10^ There is no record of previous studies on anaemia in pregnancy in Eswatini. According to the World Health Organization (WHO), anaemia amongst pregnant women is a significant health problem in low-income countries (LICs).^11^ The WHO has defined anaemia in pregnancy as haemoglobin levels of less than 11 g/dL.^5,12^ The different haemoglobin levels for each anaemia class during pregnancy are 10.0 g/dL – 10.9 g/dL for mild, 7 g/dL – 9.9 g/dL for moderate and less than 7 g/dL for severe anaemia.^13^ In addition, sub-Saharan Africa (SSA) has an estimated prevalence of 56.0%, compared with the 22.0% of high-income countries.^14,15^

In LICs, anaemia in pregnancy significantly contributes to maternal and infant morbidity and mortality.^16^ Anaemia in pregnancy is considered a risk factor for the poor pregnancy outcome, resulting in life-threatening complications for both mother and foetus.^12,14^ Foetal consequences include stillbirths, low weight, intrauterine growth restriction, premature babies, perinatal mortality and neonatal sepsis.^17,18^ In late pregnancy, anaemia results in poor foetal iron stores, which can irreversibly affect the brain and neurotransmitters in the foetus and postnatal babies, leading to developmental disorders.^19^ Effects of anaemia during pregnancy may be related to its severity, for example, mild anaemia may not affect the current pregnancy but may reduce maternal iron stores and affect subsequent pregnancies.^7^ In addition, anaemic pregnant women are more prone to many complications, including decreased work productivity, increased risk of cardiac diseases, preterm labour and delivery, postpartum haemorrhage, impaired immune function and maternal mortality.^7,11^ Anaemia during pregnancy has been estimated to account for 23% of the indirect causes of maternal deaths in developing countries.^6,19^

Moreover, several factors have been identified as contributing factors of anaemia amongst pregnant women. Iron deficiency is the most common cause; it is usually accompanied by a deficiency of other nutrients.^8,13^ Around 40% of women begin their pregnancy with decreased iron stores, which becomes insufficient to meet the increased iron needs during pregnancy.^2,20^ Other factors include physiological haemodilution, underlying inflammatory conditions and malnutrition, leading to insufficient vitamins, proteins, iron and iodine.^11,13^ In addition, in SSA, infections such as malaria, helminths and human immunodeficiency virus (HIV) also contribute to anaemia in pregnancy.^13,21^ Despite the broader scope of the problem, little research data have been explored about the severity of anaemia at antenatal care clinic (ANC) in this study area; hence, there is a need to identify and treat anaemia to avoid its complications in pregnancy.^1,11^ Furthermore, assessing the different factors contributing to anaemia in pregnancy is essential for effective anaemia management during pregnancy and provides the necessary information for planning and policymaking.^7,9^

## Methods and materials

### Study design

This study was an analytical cross-sectional study, which was conducted for the purpose of the collection of data on the socio-demographics, sexual reproductive, nutritional status, prevalence of anaemia (through testing of haemoglobin levels, which were measured at a single point) and associated risk factors amongst pregnant women who attended ANC clinic at the three study sites.

### Study setting

Eswatini is located in the southern part of Africa. The study was conducted in three randomly selected health facilities in Eswatini, namely Mankayane, Raleigh Fitkin Memorial (RFM) and Mbabane Hospital. Mankayane is located in the Western subregion of Manzini, and it serves as a referral hospital to the 14 clinics in its catchment area. Raleigh Fitkin Memorial is a regional referral and teaching hospital in the hub of the Manzini region. Mbabane Hospital is the country’s National Referral Hospital, which is situated in the capital city of Eswatini, Mbabane, in Hhohho region. In Eswatini, all ANC services in hospitals are offered in public health unit departments, which deal with Maternal and Child Health services, amongst others. These sites were easily accessible to the greater population of Eswatini.

### Study population

The study participants were pregnant women aged 15–49 years, attending ANC in the three healthcare facilities from January 2021 to March 2021. The women were recruited from these three healthcare facilities (Mankayane, RFM and Mbabane Hospital) in Eswatini. All pregnant women attending ANC in these three healthcare facilities, meeting the inclusion criteria and providing written informed consent were included. All women who were not pregnant, were above the reproductive age or who had a previous history of blood transfusion (in the last six months), were not included in the study.

### Sample size

To estimate the prevalence of anaemia, assuming 95% confidence and an acceptable margin of error of 5% and maximum variability, that is, 50% (given unknown prevalence), a sample size of 384 participants was required. The sample size was further increased by a margin of 10% to account for potential refusal and multiplied by a design effect (D) of 1.3 and the final sample size of the study was 550 pregnant women.

### Sampling strategy

The recruitment of participants was performed using non-probability sampling, where participants were recruited as they came in and as long as they met the inclusion criteria. Written informed consent was obtained from the participants and enrolment was equally offered to the participants irrespective of culture, religion, race and social class. Trained research assistants explained the study aims and procedures to the women willing to participate whilst waiting to receive health services. As part of obtaining informed consent, potential risks or possible discomforts associated with participating in the study were discussed. After obtaining written informed consent, the recruitment of participants began, which were all pregnant women aged 15–49 years seeking ANC services from the study sites. Recruitment continued until the desired sample size was reached. All women who met the criteria were recruited to the study. Those who agreed to participate were assigned an anonymous participant identification number (e.g. C01–P001 for Mankayane Government Hospital; Patient number 1). There was no linkage with the patient’s name and other personally identifiable information with the study’s findings and results but only through the participant identification number.

### Data collection

A face-to-face interview using a structured questionnaire was used by research assistants to collect the relevant information in a consultation room where privacy was considered. Prior to data collection, the research assistants were briefly trained on how to use the tool and the importance of confidentiality. The questionnaire was written in English and Siswati and those not able to read and write were assisted by the research assistants. The questionnaire included sections on socio-demographic characteristics, relevant obstetric data, nutritional data and medical history. Data were entered and stored into Statistical Package for Social Sciences (SPSS) version 27. Primary data were collected for this study. The specimens used to obtain haemoglobin results were processed in the laboratories of the same hospitals (Mankayane, RFM and Mbabane Hospital). These were collected through venipuncture by the laboratory staff, as part of the routine ANC services. The specimen used for analysis was an ethylenediaminetetraacetic acid (EDTA) tube, which was processed on a haematology analyser (Act 5 Diff, Beckman Coulter), according to the standard operating procedures. The haemoglobin result from the full blood count was used to determine whether the patient was anaemic or not, using the WHO criteria of haemoglobin concentration of less than 11 g/dL in pregnant women.^5,12^ The degree of severity of anaemia in pregnancy was classified into three categories:

- Mild anaemia: 10.0 g/dL – 10.9 g/dL
- Moderate anaemia: 7.0 g/dL – 9.9 g/dL
- Severe anaemia: less than 7.0 g/dL.^13^

### Quality control

Questionnaires were pre-tested prior to the actual data collection. The researcher conducted a two-day piloting of the questionnaires in two sites, which did not form part of the study. Ten questionnaires were used per site to conduct face-to-face interviews and these were evaluated and restructured accordingly. The collected data were checked for consistency and completeness daily. All procedures and methods were performed in accordance with the relevant guidelines, regulations and standard operating procedures.

### Statistical analysis

Data were exported from SPSS version 27 and analysed using the STATA version 15.0 (Stata Corp. College Station, Texas, United States [US]). Data were checked for possible errors and any missing values before analysis. Descriptive and inferential statistics were used for analysis. Categorical variables were summarised using frequencies and proportions, whilst continuous variables were presented using mean and standard deviation. To identify factors associated with the outcome of interest, a bivariate logistic regression analysis was performed for each independent variable and crude odds ratio with 95% confidence intervals was obtained. The strength of statistical association was measured by adjusted odds ratio and 95% confidence intervals.

### Ethical considerations

An application for full ethical approval was made to the Eswatini Health and Human Research Review Board and ethical consent was received on 18 August 2020, with ethics approval number SHR264/2020. Another application was made to the University of KwaZulu-Natal Biomedical Research Ethics Committee and ethical consent was received on 05 January 2021, with ethics approval number BREC/00002158/2020. The ethical committees approved the form used to obtain written informed consent from the participants before participating in the study. Written informed consent was obtained from all individual participants involved in the study. The study’s purpose, objectives and procedures were explained to the patients before signing the consent form. Therefore, all pregnant women aged 15–49 years seeking ANC services from the study sites were eligible for the study. According to the Eswatini national guidelines for HIV testing and counselling, the consenting age is 12 years.^22^ All procedures performed in studies involving human participants were in accordance with the ethical standards of the institutional and national research committee and with the 1964 Helsinki Declaration and its later amendments or comparable ethical standards.

## Results

### Characteristics of the study population

A total of 550 pregnant women aged 15–49 years were enrolled in the study from January to March 2021 from three randomly selected hospitals (Mankayane, RFM, Mbabane) in Eswatini. The data collected were complete: all the 550 participants had a full data set. The mean (± standard deviation [s.d.]) age for the enrolled women was 27.2 (± 6.4) years (Table 1). The highest number of the participants (29.8%) were of the age group 25–29 years, 68.4% participants had high school level education and 61.8% were unemployed. The majority (65.1%) of the women were single and 56.6% lived in urban areas. Approximately 80% of the participants’ household monthly income was less than $343.00, with many family members in the household mean of 4.0 (± 2.3). The gestational age was from 5 weeks to 40 weeks, with a mean (± s.d.) of 29.0 (± 7.74) weeks. About 36.6% of the participants were pregnant for the first time and 40.4% reported an inter-pregnancy interval of more or equal to 4 years. In this study, the inter-pregnancy interval was defined as the period when the woman was not pregnant, that is, from the date of birth of her last child to the current pregnancy. The mean (± s.d.) age at first pregnancy was 21.0 (± 3.9), ranging from 14 to 39 years. In addition, 74.9% were taking iron supplements during their current pregnancy. Regarding the weekly consumption of different foods, 40.4% of participants ate meat, 33.8% dairy products, 85.5% fruits and vegetables for more or equal to four times during the week and 51.8% participants did not eat fish. A large number of the participants were not infected with HIV (68.7%), were not on any chronic medication (66.7%) and did not have any sexually transmitted infections (98.4%) or worm infestation (99.3%) 6 months before the study. The haemoglobin results ranged from 5.9 g/dL – 15.5 g/dL with a mean (± s.d.) of 11.1 (± 1.6). In addition, 96.7% of participants had no history of anaemia in previous pregnancies, whilst 97.5% did not have anaemia 6 months before the study (Table 1).

**TABLE 1:** Characteristics of the study population (*N* = 550).

Variable	*n*	%
**Age (years)**		
15–19	75	13.6
20–24	127	23.1
25–29	164	29.8
30–34	94	17.1
35–39	79	14.4
40–44	11	2.0
**Residence**		
Rural	239	43.5
Urban	311	56.6
**Marital status**		
Single	358	65.1
Married	192	34.9
**Employment status**		
Unemployed	340	61.8
Employed	173	31.5
Self-employed	37	6.7
**Level of education**		
Never been to school	5	0.9
Primary	85	15.5
High school	376	68.4
Tertiary	84	15.3
**Household monthly income**		
Less than E5000 ($343)	434	78.9
E5000–E10 000	84	15.3
More than E10 000	32	5.8
**Gestational age (weeks)**		
1–12 (1st trimester)	18	3.3
13–26 (2nd trimester)	201	36.6
27–40 (3rd trimester)	331	60.2
**Gravidity**		
1	201	36.6
2	145	26.4
3	99	18.0
4 and above	105	19.1
**Parity**		
0	214	38.9
1	151	27.5
2	98	17.8
3	58	10.5
4 and above	29	5.3
**Number of ANC visits in this pregnancy**		
1	122	22.2
2	131	23.8
3	134	24.4
4 and above	163	29.6
**Inter-pregnancy interval (years)**		
No previous child	214	38.9
1	23	4.2
2	35	6.4
3	56	10.2
4 and above	222	40.4
**Taking Iron supplements**		
Yes	412	74.9
No	138	25.1
**Meat consumption per week**		
Does not eat	12	2.2
1	76	13.8
2	92	16.7
3	148	26.9
4 and above	222	40.4
**Fish consumption per week**		
Does not eat	285	51.8
1	177	32.2
2	54	9.8
3	19	3.5
4 and above	15	2.7
**Fruit and vegetables consumption per week**		
1	8	1.5
2	23	4.2
3	49	8.9
4 and above	470	85.5
**Dairy consumption per week**		
Does not eat	36	6.6
1	129	23.5
2	104	18.9
3	95	17.3
4 and above	186	33.8
**HIV status**		
Negative	378	68.7
Positive	172	31.3
**STI in the last 6 months**		
No	541	98.4
Yes	9	1.6
**Worm infestation in the last 6 months**		
No	546	99.3
Yes	4	0.7
**Are you on chronic medication**		
No	367	66.7
Yes	183	33.3
**Anaemia in previous pregnancy**		
No	532	96.7
Yes	18	3.3
**Anaemia 6 months before pregnancy**		
No	536	97.5
Yes	14	2.6

HIV, human immunodeficiency virus; ANC, antenatal care; E, Emalangeni; s.d., standard deviation; STI, sexually transmitted infections.

Note: Age (years): Mean ±27.2, s.d. ±6.4; Family members in household: Mean ±4.0, s.d. ±2.3; Gestational age (weeks): Mean ±28.0, s.d. ±7.7; Gestational age at first ANC: Mean ±17.4, s.d. ±6.2; Age at first pregnancy: Mean ±21.0, s.d. ±3.9; Mid upper arm circumference: Mean ±27.4, s.d. ±3.8; Haemoglobin (g/dL): Mean ±11.1, s.d. ±1.59. Haemoglobin range: 5.9 g/dL – 15.5 g/dL.

### Anaemia prevalence

The overall anaemia prevalence was 43.1%. The mild, moderate and severe cases of anaemia were 21.3%; 21.1% and 0.7%, respectively. Place of residence was statistically significant (*p* = 0.002), where anaemia prevalence was higher amongst women living in the urban area (48.9%) as compared with women living in rural area (35.6%) (Table 2). The prevalence of anaemia was highest amongst women aged 15–19 years (53.3%) and age of participants was statistically significant (*p* = 0.039). Marital status of the participants was statistically significant, with a *p*-value of 0.003. Single women had a higher prevalence of anaemia of 47.8% as compared with those who were married, 34.4%. The highest prevalence of anaemia was amongst the unemployed (44.4%) and those who had a primary level of education (48.2%), although the employment status (*p* = 0.667) and level of education (*p* = 0.416) of the participants were not statistically significant. Women who had a household monthly income of less than Eswatini Emalangeni 5000 ($343.00) had the highest anaemia prevalence of 44.9%, although household monthly income was not statistically significant (*p* = 0.234). The highest prevalence of anaemia was amongst the primigravida (45.8%). The prevalence of anaemia was 45.8% in gravida 1 and it slightly declined to 42.1% in gravida 2 participants, increased in gravida 3 (43.4%) and declined again in gravida 4 or more (39.0%). Anaemia prevalence declined from para zero (45.8%) to para two (38.8%), increased in para three (46.6%) and declined again in para four or more (34.5%). Both gravidity (*p* = 0.716) and parity (*p* = 0.632) were not statistically significant. Anaemia prevalence increased with an increase in gestational age: first trimester (33.3%); second trimester (38.3%) and third trimester (46.5%), although gestational age was not statistically significant (*p* = 0.125). Gestational age at first ANC visit (current pregnancy) was statistically significant (*p* < 0.001), where the prevalence was highest in the third trimester (80.9%) (Table 2).

**TABLE 2:** The prevalence of anaemia amongst pregnant women (15–49 years) in three hospitals in (RA.36) Eswatini (*N* = 550).

Variable	Anaemic		%	95%CI	*p*
	*N*	*n*			
Overall prevalence	550	237	43.1	38.9–47.3	-
**Age (years)**					
15–19	75	40	53.3	41.4–64.9	-
20–24	127	61	-	39.1–57.1	-
25–29	164	61	37.2	29.8–45.1	**0.039**
30–34	94	35	37.2	27.5–47.8	-
35–39	79	38	48.1	36.7–59.6	-
40–44	11	2	18.2	22.8–51.8	-
**Family members in household**					
1–4	359	160	44.6	39.4–49.9	-
5–9	171	75	43.9	36.3–51.6	**0.025**
10–14	20	2	10.0	1.2–31.7	**-**
**Residence**					
Rural	239	85	35.6	29.5–42.0	**-**
Urban	311	152	48.9	43.2–54.6	**0.002**
**Marital status**					
Single	358	171	47.8	42.5–53.1	**-**
Married	192	66	34.4	27.7–41.6	**0.003**
**Employment status**					
Unemployed	340	151	44.4	39.1–49.9	-
Employed	173	72	41.6	34.2–49.3	0.667
Self-employed	37	14	37.8	22.5–55.2	-
**Level of education**					
Never been to school	5	2	40	5.3–85.3	-
Primary	85	41	48.2	37.3–59.3	-
High school	376	164	43.6	38.5–48.8	0.416
Tertiary	84	30	35.7	25.6–46.9	-
**Household monthly income**					
Less than E5000 ($343)	434	195	44.9	40.2–49.7	
E5000–E10 000	84	31	36.9	26.6–48.1	0.234
More than E10 000	32	11	34.3	18.6–53.2	
**Gestational age (weeks)**					
1–12 (1st trimester)	18	6	33.3	13.3–59.0	-
13–26 (2nd trimester)	201	77	38.3	31.6–45.4	0.125
27–40 (3rd trimester)	331	154	46.5	41.1–52.1	-
**Gestational age at first ANC**					
1–12 (1st trimester)	121	37	30.6	22.5–39.6	-
13–26 (2nd trimester)	382	162	42.4	37.4–47.5	**< 0.001**
27–40 (3rd trimester)	47	38	80.9	66.7–90.9	-
**Age at first pregnancy**					
< 20	228	105	46.1	39.5–52.8	-
20–29	306	126	41.2	35.6–46.9	-
> 29	16	6	37.5	15.2–64.6	0.478
**Gravidity**					
1	201	92	45.8	38.7–52.9	-
2	145	61	42.1	33.9–50.5	0.716
3	99	43	43.4	33.5–53.8	-
4 and above	105	41	39.0	29.7–49.1	-
**Parity**					
0	214	98	45.8	39.0–52.7	-
1	151	64	42.4	34.4–50.7	-
2	98	38	38.8	29.1–49.2	0.632
3	58	27	46.6	33.3–60.1	-
4 and above	29	10	34.5	17.9–54.3	-
**Number of ANC visits in this pregnancy**					
1	122	53	43.4	34.5–52.7	-
2	131	58	44.3	35.6–53.2	0.525
3	134	63	47.0	38.3–55.8	-
4 and above	163	63	38.7	31.1–46.6	-
**Inter-pregnancy interval (years)**					
No previous child	214	98	45.8	39.0–52.7	-
1	23	15	65.2	42.7–83.6	-
2	35	12	34.3	19.1–52.2	0.115
3	56	24	42.9	29.7–56.8	-
4 and above	222	88	39.6	33.2–46.4	-
**Taking iron supplements**					
Yes	412	175	42.5	37.7–47.4	0.615
No	138	62	44.9	36.5–53.6	-
**Fish consumption per week**					
Does not eat	285	130	45.6	39.7–51.6	-
1	177	78	44.1	36.6–51.7	-
2	54	18	33.3	21.1–47.5	0.341
3	19	6	31.6	12.6–56.6	-
4 and above	15	5	33.3	11.8–61.6	-
**Fruit and vegetables consumption per week**					
1	8	4	50	15.7–84.3	-
2	23	7	30.4	13.2–52.9	0.628
3	49	22	44.9	30.7–59.8	-
4 and above	470	204	43.4	38.9–48.0	-
**Dairy consumption per week**					
Does not eat	36	15	41.7	25.5–59.2	-
1	129	57	44.2	35.5–53.2	-
2	104	56	53.8	43.8–63.7	0.115
3	95	39	41.1	31.1–51.6	-
4 and above	186	70	37.6	30.7–45.0	-
**HIV status**					
Positive	172	100	58.1	50.4–65.6	**<0.001**
Negative	378	137	36.2	31.4–41.3	**-**
**STI in the last 6 months**					
Yes	9	7	77.8	40.0–97.2	**0.034**
No	541	230	42.5	38.3–46.8	-
**Worm infestation in the last 6 months**					
Yes	4	1	25.0	0.6–80.6	0.463
No	546	236	43.2	39.0–47.5	-
**Are you on chronic medication**					
Yes	183	104	56.8	49.3–64.1	**< 0.001**
No	367	133	36.2	31.3–41.4	-
**Anaemia in previous pregnancy**					
Yes	18	11	61.1	35.7–82.7	0.116
No	532	226	42.5	38.2–46.8	-
**Anaemia 6 months before pregnancy**					
Yes	14	11	78.6	49.2–95.3	**0.007**
No	536	226	42.2	37.9–46.5	-

HIV, human immunodeficiency virus; ANC, antenatal care; CI, confidence interval; E, Emalangeni; STI, sexually transmitted infections.

*p*-values in bold < 0.05: statistically significant.

### Risk factors associated with anaemia in the study population

Table 3 shows the risk factors associated with anaemia in the study population. Based on the univariate analysis: increasing age (25–29 years: odds ratio [OR] = 0.52, *p* = 0.02; 40–44 years: OR: = 0.19, *p* = 0.045), being married (OR = 0.57, *p* = 0.03) and increasing number of family members in household (OR = 0.15, *p* = 0.011) were inversely associated with anaemia. Living in the urban area (OR = 1.73, *p* = 0.002), being on chronic medication (OR = 2.32, *p* < 0.001), having anaemia 6 months before pregnancy (OR = 5.03, *p* = 0.014) and being HIV positive (OR = 2.44, *p* < 0.001) were positively associated with anaemia. Gestational age at first ANC visit was also positively associated with anaemia where those whose first ANC visit was in the third trimester (OR = 9.59, *p* < 0.001) were about six-fold more likely to be anaemic as compared with those whose first ANC visit was in the second trimester (OR = 1.67, *p* = 0.021). In addition, going through the result of multivariate analysis, increasing age (25–29 years: OR = 0.43, *p* = 0.008; 40–44 years: OR = 0.15, *p* = 0.033), being married (OR = 0.61, *p* = 0.039) and increasing number of family members in household (OR = 0.18, *p* = 0.039) remained inversely associated with anaemia. Living in the urban area and having anaemia 6 months before pregnancy (OR = 1.8, *p* = 0.005 and OR = 4.64, *p* = 0.031, respectively) remained positively associated with anaemia. Gestational age at first ANC visit also remained positively associated with anaemia where those whose first ANC visit was in the third trimester (OR = 10.42, *p* < 0.001) were about six-fold more likely to be anaemic as compared with those whose first ANC visit was in the second trimester (OR = 1.62, *p* = 0.043) (Table 3).

**TABLE 3:** Risk factors associated with anaemia amongst pregnant women (15–49 years) in three hospitals in Eswatini (*N* = 550).

Risk factors	Anaemic		Unadjusted (Univariate)			Adjusted (Multivariable)		
	*n*	%	OR	95%CI	*p*	OR	95%CI	*p*
**Residence**								
Rural	85	35.6	1	ref	-	1	ref	-
Urban	152	48.9	1.73	1.23–2.45	**0.002**	1.8	1.19–2.72	**0.005**
**Marital status**								
Single	171	47.8	1	ref	-	1	ref	-
Married	66	34.4	0.57	0.40–0.82	**0.003**	0.61	0.38–0.98	**0.039**
**Age (years)**								
15–19	40	53.3	1	ref	-	1	ref	-
20–24	61	48.0	0.81	0.46–1.43	0.467	0.69	0.37–1.28	0.238
25–29	61	37.2	0.52	0.30–0.90	**0.020**	0.43	0.23–0.81	**0.008**
30–34	35	37.2	0.52	0.28–0.96	**0.037**	0.41	0.19–0.87	**0.020**
35–39	38	48.1	0.81	0.43–1.53	0.516	0.60	0.28–1.31	0.204
40–44	2	18.2	0.19	0.04–0.96	**0.045**	0.15	0.02–0.85	**0.033**
**STI in the last 6 months**								
No	230	42.5	1	ref	-	1	ref	-
Yes	7	77.8	4.73	0.97–22.99	0.054	3.9	0.63–24.06	0.143
**Are you on chronic medication**								
No	133	36.2	1	ref	-	1	ref	-
Yes	104	56.8	2.32	1.61–3.33	**< 0.001**	1.37	0.36–5.21	0.641
**Anaemia 6 months before pregnancy**								
No	226	42.2	1	ref	-	1	ref	-
Yes	11	78.6	5.03	1.39–18.24	**0.014**	4.64	1.15–18.71	**0.031**
**Family members in household**								
1–4	160	44.6	1	ref	-	1	ref	-
5–9	75	43.9	0.97	0.67–1.40	0.878	1.18	0.76–1.81	0.465
10–14	2	10.0	0.15	0.033–0.64	**0.011**	0.18	0.036–0.92	**0.039**
**HIV**								
Negative	137	36.2	1	ref	-	1	ref	-
Positive	100	58.1	2.44	1.69–3.53	**< 0.001**	2.17	0.56–8.44	0.263
**Gestational age at first ANC**								
1–12 (1st trimester)	37	30.6	1	ref	-	1	ref	-
13–26 (2nd trimester)	162	42.4	1.67	1.08–2.59	**0.021**	1.62	1.02–2.60	**0.043**
27–40 (3rd trimester)	38	80.9	9.59	4.21–21.83	**< 0.001**	10.42	4.27–25.4	**< 0.001**

HIV, human immunodeficiency virus; CI, confidence interval; OR, odds ratio; STI, sexually transmitted infections; ANC, antenata.

*p*-values in bold < 0.05: statistically significant.

## Discussion

This is the first study assessing prevalence and risk factors for anaemia in pregnancy in the Kingdom of Eswatini. The prevalence of anaemia in this study (43.1%) shows that this is a public health concern. According to the WHO, anaemia is a public health problem if its prevalence is greater than or equal to 5.0%, and a severe problem if prevalence is greater than or equal to 40.0%.^7,15^ Other researchers have reported the prevalence of anaemia during pregnancy, ranging from 32.0% to 62.2%.^15^ In SSA countries, prevalence of anaemia in pregnancy has been reported to be 57.0%.^23^ Similar prevalence of anaemia was found in South East Asian countries (48.0%),^6^ Northern Tanzania (47.0%),^15^ Kenya (40.0%),^24^ and South Africa (43.0%).^4^ Lower anaemia prevalence has been reported in Lesotho (33.2%),^25^ Botswana (34.0%),^26^ Ethiopia (36.6%) and Nigeria (37.6%).^17^ Higher prevalence of anaemia was reported in Eastern Kenya (57.0%),^27^ Pakistan (56.4%),^28^ and North East India (60.0%).^6^ The variations in prevalence of anaemia may be due to different causes of anaemia, dietary differences, population differences, study design and differences in methodology used in determining haemoglobin levels.^24,28^ Regarding the differences in severity, the similar prevalence of anaemia was reported in Northern Ghana^8^ and Egypt,^29^ where the mild and moderate anaemia cases were similar.

In this study, anaemia prevalence was highest in the age group 15–19 years (53.3%). A study in Northern Ghana reported similar findings, where anaemia prevalence in the age group 20 years and below was 51.5% compared with 47.8% in those older than 35 years.^30^ Another study in Nigerian reported the highest anaemia prevalence amongst participants in the age group of 20 years and below.^17^ High prevalence of anaemia in younger ages might have been because of the lack of awareness, poor knowledge of antenatal services and failure to seek prenatal care early and take care of themselves during pregnancy.^5,17^ However, there was a contrasting report from Ethiopia, where the highest anaemia prevalence was reported amongst participants of the age group 30–39 years.^9^ Another study in India reported the highest prevalence of anaemia in the age group 20–29 years.^6^ This might have been because of increased body weakness, multiple pregnancies, labour and being subjected to other illnesses leading to a predisposition to anaemia as age advances.^9,27^

This study reported anaemia prevalence higher amongst women living in the urban area (48.9%) than women living in rural areas (35.6%). Consistent findings were reported in a study in Southern Nigeria.^17^ This is contrary to other studies in India and Northern Ethiopia, where anaemia prevalence was high amongst pregnant women living in rural areas compared with those living in urban areas.^6,31^ This may be because of limited access to health facilities and limited resources for adequate and proper nutrition during pregnancy.^16,31,32^ Based on the finding of this study, anaemia prevalence was highest in women with a monthly household income of less than $343.00 (44.9%). These findings are consistent with a study in Ethiopia, where women from lower socio-economic classes had a higher prevalence of anaemia than those from higher socio-economic classes.^2,18^ The women from lower socio-economic status were perceived as unable to afford good quality food.^2,12^ The highest anaemia prevalence was amongst the unemployed, pregnant women (44.4%). A study in Nigeria also reported similar findings, where it stated that the participants had little or no income to buy the right food required to prevent anaemia.^17^ One study in Kenya showed contrasting results of the high prevalence of anaemia amongst the employed participants.^27^ They stated that the employed participants had no time to rest or attend ANC clinics compared with housewives.^27^

The highest anaemia prevalence was reported amongst participants who were in the third trimester of their pregnancy. Similar findings were reported in Egypt^29^ and Ghana.^8^ This might have been due to reducing iron stores as a result of increasing demand for iron for both the mother and foetus, as pregnancy progress.^2,18^ In pregnant women, late detection of anaemia increases the risk of eclampsia and post-partum haemorrhage.^5^ In the foetus and neonates, anaemia increases the risk of developmental delays, premature and stillbirths.^1,5^ There was a significant association between gestational age at first ANC and anaemia in pregnancy. In this study, those in the third trimester were about six-fold more likely to be anaemic than those in the second trimester. These findings are similar to a study reported in Ethiopia, where women in the second and third trimesters were 3.1 and 3.7 times more likely to develop anaemia than those in the first trimester.^2^ During pregnancy, physiological haemodilution occurs as the pregnancy progresses, worsening anaemia in the third trimester.^16^

The prevalence of anaemia declined from gravida one (45.8%) to gravida two (42.1%), increased in gravida three (43.4%) and declined again in gravida four or more (39.0%). There was no association between gravidity and anaemia. A study in Tanzania reported the same pattern of results, although there was no association between gravidity and anaemia^15^ and in Uganda, there was no evidence of increased risk of anaemia in multigravida as compared with primigravida.^33^ However, a study in India indicated a positive correlation between gravidity and anaemia and the same study showed an increase in anaemia prevalence as gravidity increased, which is not consistent with this study.^5^ In this study, anaemia prevalence declined from para zero (45.8%) to para two (38.8%), increased in para three (46.6%) and declined again in para four or more (34.5%). There was no association between parity and anaemia. In China, a retrospective study by Lin et al. showed no association between multiparity and anaemia.^32^ However, a study in India showed an association between anaemia and parity, where women who had given birth two or more times had the highest anaemia prevalence.^5^ This was consistent with another study in South Africa, where anaemia prevalence in primipara women was 32.7% and multi- and grand-para, 36.8%.^4^ The initial decrease in anaemia prevalence as gravidity and parity increased might have been because the small sample sizes of participants as gravidity and parity increased.^34^

This study showed that being on chronic medication was a risk for developing anaemia in pregnancy. Similarly, a study from India observed an association between anaemic pregnant women with a chronic illness in pregnancy or the recent past.^11^ Also, this study found that HIV was significantly associated with anaemia in pregnancy. In Uganda, a study showed similar findings, where pregnant women infected with HIV were twice more likely to have anaemia than their HIV-negative counterparts.^35^ The HIV infection is associated with lower serum folic acid and ferritin levels, and the use of antiretroviral drugs, especially Zidovudine, is associated with anaemia.^4,35^ In HIV-infected individuals, there is a relative deficiency of iron in the body; therefore, the body cannot effectively use iron to generate new blood cells.^11,36^

## Strengths and limitations of the study

The limitations of this study were that this was a hospital-based study, so results could only be generalised to women attending ANC and not all pregnant women in the population. In addition, the study design was cross-sectional, so it was impossible to identify and establish cause and effect relationships. Nevertheless, the findings shed some light on anaemia in pregnancy and the associated risks, and they provide a platform for further studies. The study’s strengths were that the diagnosis of anaemia was based on laboratory analysis and did not depend on clinical assessment. This is the first study on prevalence and risks associated with anaemia in pregnancy, and it gives perspective to the burden of anaemia in Eswatini. It also serves as a benchmark for further research into the role of other factors that may contribute to understanding anaemia in pregnancy.

## Recommendations

The findings of this study shed some light on anaemia in pregnancy and the associated risks, and the recommendations are as follows:

- Strengthen community health education about anaemia in pregnancy and the importance of seeking ANC services early.
- Women of childbearing age should start taking iron supplements before pregnancy, so as to decrease the chances of getting anaemia during pregnancy.
- Health education should include consequences of anaemia in pregnancy, proper nutrition, maintaining regular antenatal visits, and the importance of early diagnosis of anaemia.
- Further consultation of the anaemic patients should be carried out, in order to determine cause, which will inform treatment.
- The aim should be to prevent anaemia in pregnancy and not treatment.

## Future studies

Based on our study findings, the following future areas of research are recommended:

- A national study to get the national prevalence of anaemia in pregnancy.
- A large-scale survey to further investigate the association between the risk factors and anaemia in pregnancy.
- Follow-up on the anaemic pregnant women until after birth, to assess their birth outcomes.
- Research on factors that prevent early ANC initiation
- Investigate the effectiveness of oral iron supplementation in non-anaemic pregnant women.
- Study and identify specific aetiologies and root causes of anaemia in pregnancy.

## Conclusion

The results of this study highlight prevalence and associated risk factors for anaemia amongst pregnant women attending ANC in three hospitals in Eswatini and indicate potential factors for interference to alleviate the anaemia burden in Eswatini. The findings show that anaemia amongst pregnant women attending ANC in three hospitals in Eswatini, is associated with the place of living and gestational age at first ANC, amongst others. Also, this study concludes that the prevalence of anaemia is high amongst pregnant women in Eswatini. Furthermore, the study has provided essential information about anaemia and associated risk factors that can contribute to policy development and prevention strategies. Finally, the findings provide epidemiological knowledge about the distribution of anaemia. This is crucial to guide the continuous awareness and health education on the implication of anaemia and the importance of prevention and reducing the risk of anaemia in pregnancy.
